# Loss-of-function variants in the filaggrin gene are a significant risk factor for peanut allergy

**DOI:** 10.1016/j.jaci.2011.01.031

**Published:** 2011-03

**Authors:** Sara J. Brown, Yuka Asai, Heather J. Cordell, Linda E. Campbell, Yiwei Zhao, Haihui Liao, Kate Northstone, John Henderson, Reza Alizadehfar, Moshe Ben-Shoshan, Kenneth Morgan, Graham Roberts, Laury J.N. Masthoff, Suzanne G.M.A. Pasmans, Peter C. van den Akker, Cisca Wijmenga, Jonathan O’B. Hourihane, Colin N.A. Palmer, Gideon Lack, Ann Clarke, Peter R. Hull, Alan D. Irvine, W. H. Irwin McLean

**Affiliations:** a Epithelial Genetics Group, Division of Molecular Medicine, University of Dundee, Dundee, United Kingdom; b Biomedical Research Centre, Ninewells Hospital and Medical School, University of Dundee, Dundee, United Kingdom; c Department of Dermatology, Ninewells Hospital and Medical School, Dundee, United Kingdom; d Trinity College Dublin, College Green, Dublin, Ireland; e Division of Dermatology, Department of Medicine, McGill University Health Centre, Montreal, Quebec, Canada; f Division of Clinical Epidemiology, Department of Medicine, McGill University Health Centre, Montreal, Quebec, Canada; g Division of Clinical Immunology/Allergy, Department of Medicine, McGill University Health Centre, Montreal, Quebec, Canada; h Departments of Human Genetics and Medicine, McGill University and Research Institute of McGill University Health Centre, Montreal, Quebec, Canada; i Division of Clinical Immunology/Allergy, Department of Pediatrics, McGill University Health Centre, Montreal, Quebec, Canada; j Institute of Human Genetics, Newcastle University, Newcastle-upon-Tyne, United Kingdom; k Department of Social Medicine, University of Bristol, Bristol, United Kingdom; l University of Southampton School of Medicine and Respiratory Biomedical Research Unit, Southampton, United Kingdom; m Department of Pediatric Dermatology/Allergology, Wilhelmina's Children Hospital, University Medical Centre Utrecht, Utrecht, The Netherlands; n Department of Genetics, University Medical Center Groningen, University of Groningen, Groningen, The Netherlands; o Department of Paediatrics and Child Health, University College Cork, Cork, Ireland; p Paediatric Allergy, King's College London, London, United Kingdom; q Division of Dermatology, University of Saskatchewan, Saskatoon, Saskatchewan, Canada; r Our Lady's Children's Hospital, Crumlin, Dublin, Ireland

**Keywords:** Atopic dermatitis, filaggrin, IgE, peanut allergy, risk factor, AD, Atopic dermatitis, ALSPAC, Avon Longitudinal Study of Parents and Children, *FLG*, Filaggrin, OR, Odds ratio, SPT, Skin prick test, UK, United Kingdom

## Abstract

**Background:**

IgE-mediated peanut allergy is a complex trait with strong heritability, but its genetic basis is currently unknown. Loss-of-function mutations within the filaggrin gene are associated with atopic dermatitis and other atopic diseases; therefore, filaggrin is a candidate gene in the etiology of peanut allergy.

**Objective:**

To investigate the association between filaggrin loss-of-function mutations and peanut allergy.

**Methods:**

Case-control study of 71 English, Dutch, and Irish oral food challenge–positive patients with peanut allergy and 1000 non peanut-sensitized English population controls. Replication was tested in 390 white Canadian patients with peanut allergy (defined by food challenge, or clinical history and skin prick test wheal to peanut ≥8 mm and/or peanut-specific IgE ≥15 kUL^−1^) and 891 white Canadian population controls. The most prevalent filaggrin loss-of-function mutations were assayed in each population: R501X and 2282del4 in the Europeans, and R501X, 2282del4, R2447X, and S3247X in the Canadians. The Fisher exact test and logistic regression were used to test for association; covariate analysis controlled for coexistent atopic dermatitis.

**Results:**

Filaggrin loss-of-function mutations showed a strong and significant association with peanut allergy in the food challenge–positive patients (*P* = 3.0 × 10^−6^; odds ratio, 5.3; 95% CI, 2.8-10.2), and this association was replicated in the Canadian study (*P* = 5.4 × 10^−5^; odds ratio, 1.9; 95% CI, 1.4-2.6). The association of filaggrin mutations with peanut allergy remains significant (*P* = .0008) after controlling for coexistent atopic dermatitis.

**Conclusion:**

Filaggrin mutations represent a significant risk factor for IgE-mediated peanut allergy, indicating a role for epithelial barrier dysfunction in the pathogenesis of this disease.

An adverse immune response to peanut ingestion may be severe and is potentially life-threatening.^1^ The prevalence of IgE-mediated peanut allergy in the United Kingdom (UK) and the United States has increased significantly over the past decades^2,3^ but may now have stabilized in the UK^4^ and Canada.^5^ The prevalence of peanut allergy in preschool and school-age children is approximately 1.2% to 1.6%,^3-5^ whereas the prevalence in US adults is estimated to be 0.6%.^3^

Peanut allergy is strongly heritable, with a monozygotic twin concordance of 64% compared with 7% in both dizygotic twins^6^ and other siblings.^7^ A previously reported association with HLA class II genes^8^ has not been replicated,^9^ and the genetic basis of this disorder remains poorly understood.

Loss-of-function mutations in the filaggrin gene (*FLG*) are a strong and significant risk factor for atopic dermatitis (AD)^10^ as well as asthma in association with AD,^10^ allergic rhinitis,^11^ and elevated IgE, indicating sensitization to certain foods.^11^ The *FLG* gene encodes profilaggrin, an insoluble polyprotein that is expressed in the granular layer of the epidermis and is broken down to release filaggrin monomers in the stratum corneum.^12^ Filaggrin plays a key role in epithelial barrier function, but this protein is expressed in neither the bronchial airways nor the upper gastrointestinal tract beyond the oral mucosa^13^ or possibly the esophagus.^14^ It has been hypothesized that allergic sensitization in the atopic state occurs via either transcutaneous or transmucosal passage of allergens, a process that may be facilitated by filaggrin deficiency.^15,16^ Experimental evidence supporting a prominent role of epithelial barrier deficiency as a facilitating early event in allergic priming comes from the recent analysis of a filaggrin-deficient mouse mutant.^17^

In view of the strong association of *FLG* null mutations with atopic disease and impaired skin barrier function, we aimed to investigate their role as a risk factor for IgE-mediated peanut allergy.

## Methods

### Subjects

Seventy-one patients with peanut allergy confirmed by oral food challenge, 1000 nonsensitized controls, and the respective population controls were collected from the white European populations of England, The Netherlands, and Ireland, as follows:

#### English patients with peanut allergy

Records of thirty-five children with peanut allergy were obtained from the Avon Longitudinal Study of Parents and Children (ALSPAC).^18^ In this longitudinal birth cohort study, peanut allergy was defined by a suggestive clinical history plus a positive double-blind placebo-controlled food challenge. Subjects reported by parents as having flexural dermatitis at 2 or more time points (up to a maximum of 4) between the ages of 6 and 42 months were designated as having AD.

#### Dutch patients with peanut allergy

Twenty patients with peanut allergy were recruited from pediatric allergy clinics. Peanut allergy was defined by a positive double-blind placebo-controlled oral food challenge test to peanut. AD was defined on the basis of a dermatologist’s examination.

#### Irish patients with peanut allergy

Sixteen patients with peanut allergy were recruited from pediatric allergy clinics on the basis of a suggestive clinical history and a positive open oral food challenge test to peanut. AD was defined on the basis of a pediatrician’s examination or parental report.

#### Non–peanut-sensitized control group

ALSPAC is a longitudinal, population-based birth cohort study that originally included over 14,000 English children, as previously described.^18^ One thousand consecutive non–peanut-sensitized individuals (ie, having had a negative skin prick test [SPT] result for peanut) for whom AD status was recorded were drawn from the ALSPAC cohort as normal controls for the primary analysis. In this control group, AD was defined in the same way as for the ALSPAC patients—that is, as having flexural dermatitis at 2 or more time points between the ages of 6 and 42 months.

Demographic data for the food challenge–positive patients with peanut allergy and non–peanut-sensitized controls are shown in Table I.

#### Population control groups

Within the ALSPAC population birth cohort, relevant phenotype data were available for 6895 individuals successfully typed for the 2 most common *FLG* null mutations (R501X and 2282del4). The 6851 individuals without peanut allergy from this collection were used as an English population control group. One hundred Dutch population control samples were obtained from healthy adult blood donors attending the University Medical Centre, Groningen. Irish population controls were 100 healthy adults from the population-based Trinity Biobank control samples.

### Replication study

The replication of findings from the food challenge–proven patients was tested in a larger Canadian peanut allergy case collection, as follows:

#### Canadian patients with peanut allergy

From an established Canadian peanut allergy cohort,^19-22^ 390 white patients with peanut allergy were recruited between July 2008 and April 2009. They were defined as having peanut allergy on the basis of a positive oral food challenge (n = 25) or, in the non–food-challenged subjects, a peanut-specific IgE ≥15 kUL^−1^ (n = 65) or a SPT wheal to peanut ≥8 mm (n = 214) or both sIgE ≥15 kUL^−1^ and SPT wheal ≥8 mm (n = 86).^23-25^ A total of 249 (68%) of the patients defined by immunologic parameters had a clinical history of anaphylaxis to peanut on the basis of the consensus definition from the National Institute of Allergy and Infectious Disease/Food Allergy and Anaphylaxis Network,^26^ whereas 116 (32%) had a history strongly suggestive of type I hypersensitivity to peanut.^27^ These thresholds have gained general acceptance, and in the Canadian context, it would be ethically difficult to justify the use of a food challenge in children with values exceeding these thresholds.

The clinical and immunologic data relating to the Canadian patients with peanut allergy are summarized in Table II.

#### Canadian population controls

A total of 891 Canadian controls of white ethnicity were derived from a population collection of adult volunteers from the Toronto, Ontario, area, for whom peanut allergy status and the presence or absence of coexistent AD were unknown.

Demographic data and clinical characteristics for the replication collection are shown in Table III.

### Ethical considerations

The peanut allergy case collections were approved by the relevant local research ethics committees, and all subjects or the subjects’ guardians gave written informed consent. DNA was collected and analyzed from individuals within each of the control groups with ethical approval and written informed consent.

### Genotyping for *FLG* loss-of-function mutations

DNA was extracted from blood or saliva samples by using standard protocols. The *FLG* loss-of-function mutations that are most prevalent in the European population were genotyped by using a previously published methodology.^28^ R501X and 2282del4 had previously been typed in the ALSPAC cohort; R501X, 2282del4, R2447X, and S3247X were typed in the Dutch, Canadian, and Irish case collections in this study.

### Statistical analysis

#### Case-control analysis to test the association between filaggrin genotype and peanut allergy

The Fisher exact test and logistic regression were used to test for association between peanut allergy and the *FLG* loss-of-function mutations as a combined null genotype. The rationale for creating a combined null genotype for *FLG* is based on biochemical and immunohistochemical studies demonstrating that each *FLG* null mutation has an equivalent biological effect.^12,28^ The combined null genotype in the pooled English/ Dutch/Irish case collection used data relating to the 2 mutations (R501X and 2282del4) because these were available for all patients and the controls; the combined null genotype in the Canadian patients and controls relates to the 4 mutations (R501X, 2282del4, R2447X, and S3247X), and these 4 mutations were also analyzed in the subgroups of Dutch and Irish patients with their relevant control populations.

Analysis was performed with the statistical analysis package Stata (version 9; StataCorp LP, College Station, Tex). Logistic regression models the log odds of disease as a linear function of variables encoding allele effects at the relevant loci. A genotype variable coded as 0, 1, or 2 according to the number of mutant alleles carried at the *FLG* locus was included in the regression equation.

#### Covariate analysis to control for coexistent AD

*FLG* null mutations are strongly and significantly associated with AD,^10^ and AD is more prevalent among patients with peanut allergy than in the general population.^29,30^ We therefore investigated the role of coexistent AD as a risk factor and potential confounder in the peanut-*FLG* association by using covariate analysis in the food challenge–positive dataset. This analysis was not possible in the Canadian dataset because AD status in the control population is unknown. Covariate analysis was performed by including AD as a variable in the logistic regression equation using Stata.

## Results

### *FLG* null mutations are strongly and significantly associated with peanut allergy demonstrated by positive food challenge

Analysis of the 71 patients with peanut allergy defined by positive oral challenge, when compared with 1000 non–peanut-sensitized controls, showed a strong and significant association of *FLG* loss-of-function mutations with peanut allergy (odds ratio [OR], 5.3; 95% CI, 2.8-10.2; Fisher exact test, *P* = 3.0 × 10^−6^; Table IV).

Comparison of the English (n = 35) and Dutch (n = 20) patients with peanut allergy with their respective population controls in separate case-control analyses also showed a statistically significant association between *FLG* null mutations and peanut allergy in each population subgroup; analysis of the Irish patients with peanut allergy (n = 16) in a separate case-control analysis did not give a statistically significant result because of the small number of patients (Table V).

### *FLG* null mutations are associated with peanut allergy in a Canadian case-control study

Table VI summarizes the genotype data and association analysis for the Canadian patients and controls. The highly significant association is replicated (*P* = 5.4 × 10^−5^) with an estimated OR of 1.9 (95% CI, 1.2-2.6).

### Association between *FLG* null mutations and peanut allergy is not solely attributable to coexistent AD

Covariate analysis using both AD and *FLG* as predictors in the logistic regression model demonstrated the strong association of atopic eczema with peanut allergy in the 71 food challenge–positive patients, with an OR of 7.4 (95% CI, 4.1-13.7). However, in these same patients, having controlled for AD, the residual OR for *FLG* in association with peanut allergy was 3.8 (95% CI, 1.7-8.3; *P* = .0008).

## Discussion

The major known risk factors for IgE-mediated peanut allergy are family history of the disease and coexistent atopy.^29^ However, despite the clinical importance of peanut allergy and its strong heritability, no significant genetic risk factors have previously been confirmed by replication studies. Taken together, our experimental data from 4 populations of European origin demonstrate a strong and significant association of loss-of-function mutations within the *FLG* gene with clinically significant peanut allergy. Furthermore, we have confirmed, by using covariate analysis in the food challenge–positive patients, that this association does not simply represent a coassociation with AD. The association between *FLG* and peanut allergy is not fully accounted for by confounding with coexistent AD, because the statistically significant association of the combined *FLG* null genotype with peanut allergy persists even after controlling for AD (*P* = .0008).

The differing case collections chosen for this genetic epidemiologic study offer complementary insights into the possible link between *FLG* genotype and peanut allergy. The white European dataset had a positive result to food challenge testing that was double-blind and placebo-controlled in the English (n = 35) and Dutch (n = 20) patients and an open challenge in the Irish (n = 16) patients. Double-blind placebo-controlled oral food challenge is the gold standard in a research setting, but open or single-blind challenges are more standard clinical practice,^2,30^ particularly in children. The ALSPAC cohort is a large, unselected population birth cohort in which data have been collected on a prospective basis, providing the opportunity for a case-control study in which the controls are appropriately matched to patients and in which data on coexistent AD have been collected. The Canadian collection of patients with peanut allergy, however, provides a much greater statistical power because of the larger number of individuals with peanut allergy available for analysis. There is a striking age difference between the Canadian patients and the control population. However, because AD does not affect survival pattern, this study design would not be predicted to affect the findings or interpretation of data.

The higher OR of disease in the combined English, Dutch, and Irish dataset compared with the Canadian study may be explained by the use of a hypernormal (nonsensitized) English control population, whereas the Canadian population controls would include individuals with current or previous peanut sensitization or allergy, resulting in a lower OR. However, it is interesting to note that the estimated OR is 3.2 to 3.5 (95% CI, 1.0-11.7) in each of the population subgroups when they are compared with their respective unselected population controls (Table V). Alternatively, or in addition, there may be unidentified *FLG* null mutations within the white Canadian population that have not been included in the statistical analysis, therefore possibly leading to an underestimation of the *FLG* association. We limited our analysis in these large studies to the 2 or 4 most common *FLG* mutations and are therefore likely to have underestimated the total contribution of *FLG* null alleles to this disease association. We limited our analyses to these selected mutations because the *FLG* gene is large and its DNA sequence is highly repetitive, making genotyping technically difficult.^12^

It would be interesting and informative to dissect further the association of *FLG* status with AD, food sensitization, and allergy. However, the separation of AD, food allergy, and food sensitization data is complex and challenging because these phenotypes show considerable overlap. Furthermore, the definition of peanut allergy in the Canadian patients necessarily includes peanut sensitization data, and our analysis is limited because data on AD and food allergy phenotypes are not available for the Canadian control population. The analysis of *FLG* genotype and peanut allergy with AD as a confounding factor was therefore limited to the food challenge–positive patients and nonsensitized controls.

The estimated OR for *FLG* as a risk factor for peanut allergy is 5.3 (95% CI, 2.8-10.2) in the pooled analysis of English/Dutch/Irish food challenge–positive patients for whom 2 *FLG* loss-of-function mutations were assessed; the equivalent OR is 1.9 (95% CI, 1.4-2.6) in the Canadian collection (Table VI), 3.5 (95% CI, 1.1-11.4) in the Dutch collection, and 3.3 (95% CI, 1.0-11.7) in the Irish collection (Table V), for whom 4 *FLG* null mutations were assessed. A total of over 20 *FLG* null mutations have now been identified within populations of European ancestry. In the Irish population, the most intensively studied white European population with respect to *FLG*, R501X and 2282del4 together represent about 66% of the *FLG* mutations; adding R2447X and S3247X covers about 95% of the known *FLG* mutations.

The prevalence of food challenge–positive peanut allergy within this subset of the 1989 to 1990 ALSPAC birth cohort is 0.6%, consistent with other published data.^2^ The proportion of these patients carrying 1 or more *FLG* null mutations (associated with an estimated OR of 3.2) is 20.0% (Table V). Similarly, in the Canadian study, 19.2% of the patients with peanut allergy carry 1 or more *FLG* null mutations, with an estimated OR of 1.9 (Table VI). These data demonstrate the striking contribution of a single gene in the etiology of a complex trait resulting from multiple genetic and environmental effects.

The mechanisms by which filaggrin deficiency in the skin may lead to AD and other atopic disorders have been debated extensively.^15,31,32^ It is proposed that impairment of the epidermal barrier allows the penetration of allergens and irritants, resulting in local inflammation as well as a systemic atopic response. There is experimental evidence in support of this hypothesis from mouse models^17,33-35^ as well as clinical epidemiologic studies showing that *FLG* null mutations are a risk factor for asthma only in the subtype of asthma that is associated with AD.^10^ Similarly, it has been suggested that peanut sensitization may result from transcutaneous exposure to peanut allergens in the environment^36^ or in topical preparations,^29^ particularly because early-onset AD is a risk factor for peanut allergy.^29^ This contrasts with the oral route of exposure, which is thought to be tolerogenic.^37^ However, intestinal permeability is known to be increased amongst some patients with AD,^38^ and it has been suggested that intestinal permeability may be an additional route of allergen penetration in peanut allergy and other food allergies.^39^ The extent and functional significance of filaggrin expression in the gastrointestinal tract below the oral mucosa is currently unknown but warrants further investigation. This study focused on the association between *FLG* loss-of-function mutations and peanut allergy; the possibility of association with other food allergies has not been addressed and also warrants further investigation. One previous study of *FLG* genotype and sensitization to foods was conducted as a subgroup analysis within a German cohort study on asthma risk.^40^ This analysis showed a strong synergistic interaction between the *FLG-*null alleles and early food sensitization in the disease transition from eczema to asthma (relative excess risk, 2.64) but no significant association of *FLG* status with food sensitization *per se* in 185 children with AD. Although the subgroup sample size was rather small, these contrasting data may reflect differences in the pathogenesis of food sensitization versus food allergy as well as possible differences in the pathogenesis of allergy to different foods.

The well recognized strong association of AD with peanut allergy is demonstrated in the results of covariate analysis, in which eczema shows an estimated OR of 7.4 compared with the *FLG* estimated OR (after accounting for AD) of 3.8. Hence, AD is a stronger independent risk factor for peanut allergy than *FLG* null mutations. However, it is interesting and noteworthy that the *FLG* genotype effect is not solely accounted for by a coassociation with AD, a finding that contributes to the debate outlined regarding the routes and mechanisms of allergic sensitization. It is possible that the association of *FLG* loss-of-function mutations persists because of incorrect assignment of patients with AD to the non-AD subgroup (ie, incomplete case ascertainment of AD on the basis of these definitions). The case definition used for AD, flexural dermatitis reported by parents on at least 2 occasions (in the ALSPAC cohort), could possibly have failed to include children with mild, localized, or transient disease in the AD case collection. However, the high prevalence of coexistent AD in the ALSPAC cohort, at 27.1% in subjects without peanut allergy (Table III) and 27.6% in the cohort as a whole, in comparison with published UK studies,^41^ suggests that few patients have been missed by this case definition. Absence of established AD according to the case definitions used in these studies does not preclude a significant epidermal barrier defect in filaggrin-deficient individuals. Murine models of filaggrin deficiency have shown enhanced percutaneous allergen sensitization in 3 studies.^17,34,35^ These mice appear to have an inherent epithelial barrier defect that facilitates allergen priming in the absence of clinical evidence of cutaneous inflammation.

Here we have shown that *FLG* mutations confer a risk for peanut allergy in the absence of clinical evidence of AD. In contrast, although *FLG* null alleles confer a significant risk of asthma, with a reproducible OR of 1.8, this effect is confined to those with pre-existent or coexistent AD.^10^ The mechanistic pathways through which *FLG* null alleles contribute risk to these related but distinct atopic phenotypes are as yet unclear.

In conclusion, *FLG* null mutations not only represent a highly significant genetic risk factor for AD but also are the single most significant genetic risk for peanut allergy that has been identified to date. The robustness of this association is demonstrated by replication within 3 populations— English, Dutch, and Canadian—plus an association close to significance in an Irish collection. The association of *FLG* mutations with peanut allergy adds further insight into the pathogenesis of the atopic state and the critical role of epithelial barrier function in health and disease.

**Key messages**

• Loss-of-function variants in the filaggrin gene are strongly and significantly associated with peanut allergy.
• The association of filaggrin mutations with peanut allergy cannot fully be accounted for by the confounding effect of coexistent AD.
• Filaggrin mutations are a novel risk factor for IgE-mediated peanut allergy, indicating a role for epithelial barrier dysfunction in the pathogenesis of this disease.

## Figures and Tables

**Table I:** Demographic data and clinical characteristics of 71 food challenge–positive patients with peanut allergy and a nonsensitized control group

Patients and controls	No. of individuals	Age (y), range (mean)	Male sex, n (%)	Coexistent AD, n (%)
Patients with peanut allergy from an English population birth cohort	35	Cohort recruited at birth and followed up for ≥7 y	19 (54.3)	16 out of 22 for whom AD data are available (72.7)
Dutch patients with peanut allergy	20	3-14 (7.5)	14 (70.0)	17 (85.0)
Irish patients with peanut allergy	16	1-18 (10.5)	7 (43.8)	10 (62.5)
Total patients with peanut allergy	71	Not applicable	40 (56.3)	43 (74.1)
Non–peanut-sensitized controls from English population birth cohort	1000	Cohort recruited at birth and followed up for ≥7 y	544 (54.4)	270 (27.0)

The English population birth cohort is ALSPAC; food challenges in the English and Dutch case collections were double-blind placebo-controlled; in the Irish patients, peanut allergy was confirmed by an open oral food challenge; % coexistent AD refers to the proportion of patients for whom AD data were available.

**Table II:** Clinical and immunologic parameters in Canadian patients with peanut allergy

Case definition		n
Positive oral food challenge test to peanut		25
History of anaphylaxis to peanut^26^ (n = 249)	Peanut-specific IgE ≥15 kU/L SPT to peanut ≥8 mm Peanut-specific IgE ≥15 kU/L and SPT to peanut ≥8 mm	48 141 60
History suggestive of a type I hypersensitivity reaction to peanut^27^ (n = 116)	Peanut-specific IgE ≥15 kU/L SPT to peanut ≥8 mm Peanut-specific IgE ≥15 kU/L and SPT to peanut ≥8 mm	17 73 26
Total		390

A total of 390 patients with peanut allergy were drawn from an established Canadian case collection, defined as having peanut allergy on the basis of a positive oral food challenge (n = 25) or, in the non–food-challenged subjects, a peanut-specific IgE ≥15 kUL^−1^ (n = 65) or an SPT wheal to peanut of ≥8 mm (n = 214) or both sIgE ≥15 kUL^−1^ and SPT wheal ≥8 mm (n = 86).

**Table III:** Demographic data and clinical characteristics of the Canadian replication study

Patients and controls	No. of individuals	Age (y), range (mean)	Male sex, n (%)	Coexistent AD, n (%)
White Canadian patients with peanut allergy	390	0-21 (9.5)	239 (61.3)	286/383 (69.5)
White Canadian population controls	891	23-77 (57.5)	281 (31.5)	Unknown

AD is defined according to parental report. Data were available in 383 Canadian patients; 266 reported AD, of whom 257 reported that a physician had made or confirmed the diagnosis of AD; % coexistent AD refers to the proportion of patients for whom AD data were available.

**Table IV:** Genotyping results and statistical analysis of filaggrin loss-of-function mutations in 71 food challenge–proven patients with peanut allergy and 1000 non–peanut-sensitized controls

Genotypes and statistical tests	Patients (n)	Controls (n)
No *FLG* mutations detected (homozygous wild-type)	59	963
One wild-type and 1 *FLG* mutant allele (heterozygous)	10	37
Two *FLG* loss-of-function mutations (homozygous or compound heterozygous)	2	0
Total	71	1000
Proportion of individuals carrying *FLG* loss-of-function mutations (%)	16.9	3.7
Fisher exact test	*P* = 3.0 × 10^−6^	
OR (95% CI)	5.3 (2.8-10.2)	

This combined population dataset relates to the 2 most prevalent *FLG* loss-of-function mutations (R501X and 2282del4) in English (n = 35), Dutch (n = 20), and Irish (n = 16) patients with peanut allergy demonstrated by oral food challenge. The designations homozygous wild-type, heterozygous, and 2 null mutations represent the combined null genotype results for the 2 available mutations; individuals with 2 null mutations may be homozygous for an *FLG* null allele or compound heterozygous, having 2 different *FLG* null alleles.

**Table V:** Genotyping results and statistical analysis of filaggrin loss-of-function mutations in patients with peanut allergy and matched controls from English, Dutch, and Irish populations

Genotypes and statistical tests	English (ALSPAC)		Dutch		Irish	
Patients (n)	Controls (n)	Patients (n)	Controls (n)	Patients (n)	Controls (n)
No *FLG* mutations detected (homozygous wild-type)	28	6368	17	95	13	93
One wild-type and 1 *FLG* mutant allele (heterozygous)	7	480	1	5	2	7
Two *FLG* loss-of-function mutations (homozygous or compound heterozygous)	0	3	2	0	1	0
Total	35	6851	20	100	16	100
Proportion of individuals carrying *FLG* loss-of-function mutations (%)	20.0	7.1	15.0	5.0	18.8	7.0
Fisher exact test	*P* = .0251		*P* = .0335		*P* = .0640	
OR (95% CI)	3.2 (1.4-7.2)		3.5 (1.1-11.4)		3.3 (1.0-11.7)	

The ALSPAC is a longitudinal, population-based birth cohort study.^18^ This English population was screened for the 2 most common *FLG* null mutations (R501X and 2282del4). In the Dutch and Irish patients and controls, the 4 most prevalent *FLG* loss-of-function mutations were assayed (R501X, 2282del4, R2447X, and S3247X). The designations homozygous wild-type, heterozygous, and 2 null mutations represent the combined null genotype results for the available mutations in each population.

**Table VI:** Genotyping results and statistical analysis of filaggrin loss-of-function mutations in a case-control study in the Canadian population

Genotypes and statistical tests	Patients (n)	Controls (n)
No *FLG* mutations detected (homozygous wild-type)	315	793
One wild-type and 1 *FLG* mutant allele (heterozygous)	66	94
Two *FLG* loss-of-function mutations (homozygous or compound heterozygous)	9	4
Total	390	891
Proportion of individuals carrying *FLG* loss-of-function mutations (%)	19.2	11.0
Fisher exact test	*P* = 5.4 × 10^−5^	
OR (95% CI)	1.9 (1.4-2.6)	

Patients and controls were screened for the 4 most prevalent *FLG* loss-of-function mutations (R501X, 2282del4, R2447X, and S3247X). The designations homozygous wild-type, heterozygous, and 2 null mutations represent the combined null genotype results for the 4 available mutations.
